# From ARFID to Binge Eating: A Review of the Sensory, Behavioral, and Gut–Brain Axis Mechanisms Driving Co-Occurring Eating Disorders in Children and Adolescents with Autism Spectrum Disorder

**DOI:** 10.3390/nu17233714

**Published:** 2025-11-26

**Authors:** Marta Kopańska, Izabela Łucka, Maria Siegel, Julia Trojniak, Maria Pąchalska

**Affiliations:** 1 Department of Medical Psychology, Faculty of Medicine, University of Rzeszów, al. Tadeusza Rejtana 16C, 35-959 Rzeszów, Poland; 2 Department of Developmental Psychiatry, Psychotic Disorders and Old Age Psychiatry, Medical University of Gdansk, 80-210 Gdansk, Poland; 3 Student Research Club “Reh-Tech”, Faculty of Medicine, University of Rzeszów, al. Tadeusza Rejtana 16C, 35-959 Rzeszów, Poland; 4 Department of Neuropsychology and Neurorehabilitation, Andrzej Frycz Modrzewski Krakow University, 30-705 Cracow, Poland

**Keywords:** neurodevelopmental disorders, eating disorders, gut microbiota, gut–brain axis, food selectivity, ARFID, anorexia nervosa, orthorexia nervosa

## Abstract

**Background:** Autism spectrum disorder (ASD) constitutes a heterogeneous neurodevelopmental condition frequently accompanied by considerable disturbances in feeding behavior and nutritional balance. These difficulties arise from complex and multifactorial mechanisms, exerting a significant impact on physical health, metabolic homeostasis, and psychosocial functioning. The present review aims to provide a critical synthesis of current evidence regarding the underlying biological and behavioral mechanisms of feeding difficulties in ASD and to delineate the spectrum of comorbid eating disorders within this population. **Methods:** A narrative review of the peer-reviewed scientific literature was undertaken, emphasizing studies investigating the interrelationship between ASD and nutritional functioning in pediatric and adolescent populations. Particular focus was placed on research exploring sensory processing abnormalities, gut microbiota alterations, and the clinical manifestation of eating disorders in individuals with ASD. **Results:** The analysis revealed that sensory hypersensitivity, behavioral inflexibility, and disturbances within the gut–brain axis constitute principal determinants of atypical eating patterns in ASD. Individuals on the autism spectrum frequently exhibit pronounced food selectivity, neophobia, and symptoms consistent with Avoidant/Restrictive Food Intake Disorder (ARFID). Furthermore, an increased prevalence of anorexia nervosa and orthorexia nervosa has been documented, likely reflecting shared cognitive and behavioral features with ASD. “Emotional eating” tendencies and a marked preference for high-caloric, energy-dense foods—often potentiated by psychopharmacological treatment and reduced physical activity—further contribute to an elevated risk of overweight and obesity. **Conclusions:** Children and adolescents with ASD display a bimodal distribution of body mass, encompassing both undernutrition and obesity, indicative of a multifaceted interplay among sensory, behavioral, cognitive, and metabolic determinants. A comprehensive understanding of this heterogeneity is crucial for the development of individualized, evidence-based interventions integrating nutritional management with behavioral and psychotherapeutic approaches.

## 1. Introduction

Autism spectrum disorder (ASD) is classified as a neurodevelopmental condition, marked by enduring challenges in communication, cognitive functioning, and social interaction, as well as by restricted and/or repetitive patterns of behavior, interests, or activities that typically manifest in early childhood [1]. Although various psychological assessment tools exist, and emerging research continues to explore objective neurophysiological biomarkers, the diagnosis ultimately relies on clinical evaluation [2,3].

In the DSM-5 diagnostic classification, as in ICD-11, two core symptom domains for ASD have been distinguished: deficits in social communication and social interaction, and restricted, repetitive patterns of behavior, interests, or activities (stereotypies and rituals) [1,4]. Although symptoms may manifest in early childhood, modern diagnostic classifications have abandoned a specific age criterion, as symptoms may go unnoticed until social demands exceed the child’s capabilities, or they may be masked by learned coping strategies [5].

The etiology of autism spectrum disorder (ASD) remains incompletely elucidated. Current evidence strongly supports a substantial genetic contribution to its pathogenesis [6]. However, environmental influences also play an important role. ASD is viewed as a result of a multifactorial interaction between genetic predisposition and environmental influences, wherein genetic susceptibility constitutes a foundational determinant that may be modulated through epigenetic mechanisms. Additionally, sociocultural trends in Western and Central European populations, such as substance use risks or the pressure for “clean” eating, may interact with autistic cognitive rigidity to modify behavioral phenotypes [7,8,9].

The prevalence of ASD in the general population has exceeded 1%, and in the developmental age group, it is estimated to be around 1 in 54 children [10]. In a 2020 study conducted by the Centers for Disease Control and Prevention in the USA among 8-year-old children, the prevalence of ASD was estimated at an average of 27.6 per 1000, which corresponds to one in 36 children, with the prevalence among boys being 3.8 times higher than among girls [11]. The observed increase in prevalence may be attributed to improved diagnostic methods, including the exploration of objective neurofunctional assessments, as well as greater awareness among healthcare professionals, educators, and the general public [12]. High-functioning individuals with ASD often use strategies to mask their social deficits, known as “social camouflaging”. Although camouflaging occurs in both sexes, it is presumed to be more frequently and effectively used by women [7,13].

Autism spectrum disorder encompasses a diverse group in terms of intellectual abilities, social functioning, and eating habits. Patients in this group often experience co-occurring neurological, psychiatric, and somatic anomalies. Additionally, specific eating habits, such as food neophobia, defined as a reluctance to try new foods that negatively impacts dietary quality and health status, significantly contribute to these risks. Coupled with challenges in self-care, self-destructive behaviors, and the side effects of pharmacotherapy, these factors predispose individuals with ASD to chronic diseases and body weight abnormalities [14].

Clinical and epidemiological data indicate a significantly elevated risk of co-occurrence between Autism Spectrum Disorder and Eating Disorders (EDs) [15]. Although eating disorders constitute a broad diagnostic category, defined as persistent disturbances of eating or eating-related behaviors that result in significant impairment of physical health or psychosocial functioning, a distinct overrepresentation of restrictive subtypes is observed within the ASD population [15,16]. It is estimated that atypical eating behaviors may affect over 70% of children with ASD, compared to less than 5% in the neurotypical population [16]. Concurrently, difficulties in emotion regulation and sensory dysfunctions may predispose individuals to Binge-Eating Disorder (BED). This mechanism may be associated with the use of food as a strategy for affect regulation (“emotional eating”) or the pursuit of sensory stimulation, distinguishing the etiology of these disorders in ASD from classical models driven by body image concerns [15].

In recent years, there has been growing interest in the gut–brain axis as a multidirectional communication channel integrating the nervous, endocrine, and immune systems [17]. Currently, this concept is expanded to the “microbiome–gut–mucosa–immune–brain axis”, highlighting the pivotal role of Neuro-Immuno-Gastroenterology in the pathogenesis of neurodevelopmental disorders [18]. In ASD, a selective diet frequently leads to dysbiosis, which may compromise the integrity of the intestinal barrier (“leaky gut”) and facilitate the translocation of bacterial metabolites, such as lipopolysaccharides, into the bloodstream [18]. The resulting systemic inflammatory state influences neurotransmission and emotional regulation, creating a “vicious cycle” mechanism that secondarily exacerbates behavioral symptoms [18].

The extant literature, including recently published original studies, has provided crucial data on specific aspects of nutritional problems in ASD [19,20]. Despite the value of this information, the literature still lacks a synthesis that integrates these distinct domains, including sensory, behavioral, and metabolic factors, with the role of the gut microbiota and the gut–brain axis. Crucially, no review has yet elucidated how these complex mechanisms contribute to the full spectrum of co-occurring, formally diagnosed eating disorders—ranging from restrictive disorders such as ARFID and Anorexia Nervosa to emotional and binge-eating patterns leading to obesity.

This review aims to provide a critical synthesis of these three domains, proposing the thesis that sensory hypersensitivity and behavioral rigidity in ASD lead to restrictive eating patterns, which in turn induce gut dysbiosis. This dysbiosis, through neuroinflammation and dysregulation of the gut–brain axis, secondarily affects emotional regulation and cognitive functions, predisposing individuals to the development of the full spectrum of eating disorders, ranging from ARFID to binge-eating disorders.

## 2. Methods

Given the heterogeneous and interdisciplinary nature of the research question, which encompasses neurodevelopmental psychiatry, sensory processing, gastroenterology, and microbiology, a narrative review methodology was adopted. This approach allows for the broad synthesis of diverse evidence streams required to construct an integrated pathogenetic model, a task often constrained by the rigid inclusion criteria of systematic reviews. The review aims to critically evaluate current theoretical frameworks, identify mechanistic overlaps, and highlight gaps in the existing literature to inform future clinical and research directions. To ensure rigor and minimize bias, a structured search strategy and defined eligibility criteria were implemented as detailed below.

### 2.1. Literature Search Strategy

A literature search was conducted across international scientific databases, including PubMed, Scopus, and Google Scholar, covering articles published between January 2010 and 18th November 2025. To ensure a systematic approach, search terms were organized into three logical clusters:

1. Population (e.g., “Autism Spectrum Disorder”, “ASD”, “Children”, “Adolescents”);
2. Condition/Outcome (e.g., “Eating Disorders”, “ARFID”, “Anorexia Nervosa”, “Food Selectivity”, “Binge Eating”);
3. Mechanisms (e.g., “Gut Microbiota”, “Gut-Brain Axis”, “Sensory Processing”).

Boolean operators “and” and “or” were employed to combine these terms effectively. Additionally, reference lists of identified relevant reviews and meta-analyses were manually screened to identify further eligible studies (snowballing method).

An initial database search identified numerous potential articles. After the removal of duplicates, the remaining titles and abstracts were independently reviewed by two authors for compliance with the inclusion criteria. Subsequently, the full texts of articles deemed potentially relevant were assessed for final eligibility. Any discrepancies in assessment were resolved through discussion and consensus. The reference lists of the identified articles were also screened to find additional relevant publications. Ultimately, 86 studies met the criteria and were included in the narrative synthesis.

### 2.2. Inclusion and Exclusion Criteria

Peer-reviewed scientific articles, including original research, systematic reviews, and meta-analyses focusing on the pediatric and adolescent population diagnosed with ASD, were included in the review. The primary criterion was the investigation of the relationship between ASD and at least one of the following areas: eating habits, nutritional status, gastrointestinal symptoms, gut microbiota, sensory mechanisms related to eating, and co-occurring eating disorders.

Excluded from the analysis were: conference proceedings, abstracts, dissertations, letters to the editor, commentaries, and articles that had not undergone a peer-review process. Studies focusing exclusively on adult populations or those not directly addressing nutritional topics were also omitted.

### 2.3. Data Synthesis

A total of 86 studies that met the inclusion criteria were subjected to narrative analysis and synthesis. The data synthesis process was based on thematic analysis—consistent with the Braun and Clarke approach [21]. Information from the qualified articles was systematically coded and categorized based on the main objectives of the review to identify key domains: (1) sensory mechanisms, (2) behavioral factors, and (3) the role of the gut–brain axis and microbiota. This approach allowed for an integrated, narrative synthesis of the results rather than a mere compilation. The main emphasis was placed on creating a cohesive description of the mechanisms underlying feeding problems in ASD (sensory and behavioral factors, the role of the gut–brain axis) and on presenting the clinical characteristics of the most common co-occurring eating disorders and their impact on overall health.

## 3. Sensory and Behavioral Mechanisms Driving Nutritional Selectivity

Feeding difficulties in ASD arise from a complex interplay between sensory processing abnormalities and cognitive-behavioral rigidity. Research indicates that sensory hypersensitivity, particularly within the oral domain (oral defensiveness), is a primary driver of food refusal [22,23]. This hyper-reactivity to texture, consistency, temperature, and color often co-occurs with a profound need for predictability and sameness—a core behavioral feature of ASD [24,25]. Consequently, the introduction of novel foods (neophobia) triggers anxiety and resistance, leading to a highly restrictive diet often limited to specific “safe” foods rich in carbohydrates and processed fats, while lacking essential nutrients found in fruits and vegetables [22]. These sensory-behavioral patterns also extend to oral hygiene and health. Atypical oral habits (e.g., bruxism, pouching) and self-injurious behaviors in the head and neck region, observed in up to 75% of cases with severe ASD, further complicate feeding by causing dental pathology and discomfort [14]. Thus, sensory aversion and behavioral inflexibility act synergistically to establish a restrictive eating phenotype, which serves as the behavioral starting point for the physiological dysregulation discussed below. To provide a comprehensive overview of these complex interactions, the key determinants of atypical eating patterns in ASD are summarized in Table 1.

## 4. The Gut–Brain Axis: A Biological Mediator Between Selective Diet and the Neurobiology of ASD

Selective dietary habits in ASD induce significant alterations in the gut microbiome composition, leading to a state of dysbiosis characterized by a reduction in beneficial fermentative bacteria (e.g., *Bifidobacterium*) and an overgrowth of potentially pathogenic species (e.g., *Clostridium* species) [18,26]. This dysbiosis state may compromise the intestinal epithelial barrier (“leaky gut”), facilitating the translocation of bacterial metabolites such as lipopolysaccharides (LPS) into the systemic circulation. This process is hypothesized to trigger a chronic, low-grade inflammatory response, characterized by elevated levels of pro-inflammatory cytokines (e.g., TNF-α, IL-6), which can cross the blood–brain barrier and modulate neurodevelopmental and behavioral trajectories via neuroimmune and neuroendocrine pathways [18,27].

While the “microbiome–gut–mucosal–immune–brain” axis offers a compelling mechanistic model, it is essential to interpret current findings with caution. A substantial portion of the existing literature relies on cross-sectional studies with relatively small sample sizes and high heterogeneity in methodology (e.g., differences in DNA sequencing techniques, lack of dietary control). Furthermore, most human studies demonstrate correlations rather than causation. It remains a subject of debate whether gut dysbiosis is a primary driver of ASD symptoms or a secondary consequence of the restricted “autistic diet” [20,28]. Nevertheless, bidirectional signaling is evident, where diet-induced dysbiosis exacerbates behavioral symptoms, creating a feedback loop that further entrenches restrictive eating patterns.

Specific microbial alterations driving this feedback loop include an increased abundance of *Enterobacteriaceae*, *Salmonella*, *Escherichia/Shigella* and *Clostridium XIVa* [29]. These shifts are frequently correlated with the severity of gastrointestinal symptoms, such as abdominal pain, constipation, and diarrhea, commonly reported in this population [26].

Beyond immune activation, the microbiome exerts influence via neurochemical pathways. A critical mechanism involves the stimulation of serotonin synthesis through tryptophan hydroxylation in intestinal cells. Given that polymorphisms in the serotonin transporter gene are established risk factors for ASD, dysbiosis-induced irregularities in serotonin production may directly exacerbate behavioral rigidity and mood dysregulation. The functional impact of these microbial shifts is supported by intervention studies, for instance, treatment with antibiotics such as vancomycin has been shown to temporarily alleviate behavioral difficulties. Furthermore, a recent study on fecal microbiota transplantation (FMT) in 18 children with ASD demonstrated increased bacterial diversity and concurrent improvements in both gastrointestinal and behavioral symptoms, persisting for up to eight weeks post-intervention [14].

**Figure 1:**
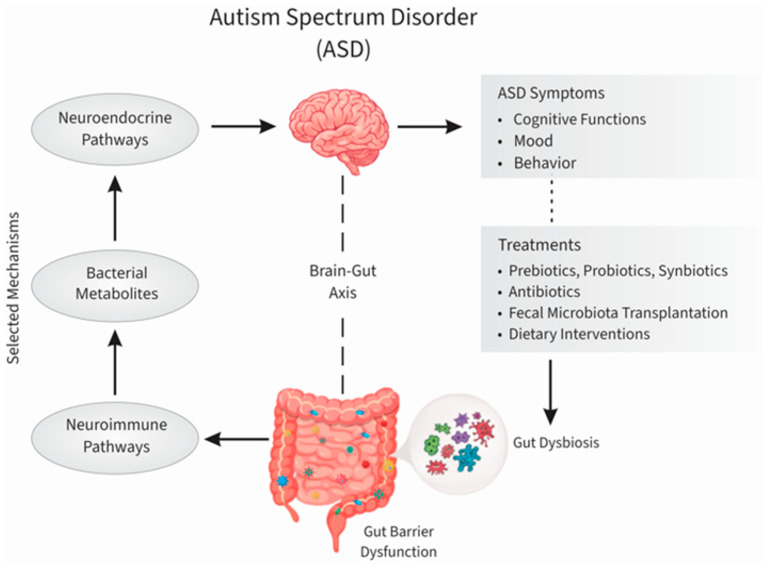
A proposed pathophysiological model of the microbiota–gut–brain axis in Autism Spectrum Disorder (ASD). The diagram delineates the bidirectional signaling pathways connecting gastrointestinal pathophysiology with neurodevelopmental outcomes. Intestinal dysbiosis and compromised barrier integrity facilitate the translocation of bacterial metabolites, activating neuroimmune and neuroendocrine cascades (e.g., cytokine production, vagus nerve modulation). These peripheral disruptions impact central nervous system function, thereby exacerbating core ASD symptomatology, including cognitive rigidity and mood dysregulation. Furthermore, the model highlights targeted therapeutic interventions—such as probiotics, prebiotics, and fecal microbiota transplantation—aimed at restoring microbial homeostasis to ameliorate these clinical manifestations. Figure created by the authors based on [14,27,28,30,31].

To address the limitations of current cross-sectional research, ongoing prospective initiatives such as the GEMMA (Genomic, Environmental, Microbiome, and Metabolomic Assessment) project aim to elucidate the longitudinal interactions between host genetics, environmental factors (e.g., birth mode, early feeding), and immune-metabolic trajectories [28]. By identifying specific metagenomic phenotypes, such studies seek to validate biomarkers for early diagnosis and shift the treatment paradigm toward targeted microbiota manipulation [28]. Collectively, the dysregulation of the oral and gut microbiota constitutes a key node in the “microbiome–gut–brain” axis, contributing to the clinical expression of ASD symptoms, as summarized in Figure 1 [14,27,28].

### Microbiota-Based Interventions: Probiotics

Recent studies highlight the potential role of microbiological interventions, such as probiotics (e.g., *Lactobacillus reuteri*, *Bifidobacterium longum*, and *Lactobacillus plantarum*), in modulating the gut microbiota and influencing behavioral responses. A double-blind, randomized, placebo-controlled trial showed that precision microbiological therapies can improve social behaviors but do not significantly alter the core symptoms of ASD. A meta-analysis of studies conducted in Italy, the United States, Taiwan, and China, which evaluated the effectiveness of probiotic supplementation in children with ASD aged 3 to 14, found no improvement in social skills, development, or overall clinical functioning, which was consistent with earlier studies [32]. However, probiotics effectively reduced gastrointestinal symptoms, suggesting their potential as a therapeutic strategy by lowering levels of 4-ethylphenylsulfate (4EPS), identified as a potential biomarker for ASD [33]. A study by Sun et al. showed that multi-strain probiotics can support the physical growth of patients by improving intestinal function [34]. These findings suggest that while probiotics may have a limited direct impact on the behavioral symptoms of ASD, they can positively affect overall health. Additionally, they appear to improve the body mass index, which influences the growth and development of children [35]. Various factors that might contribute to the poor efficacy of probiotics were analyzed, including the complex and multifactorial pathophysiological mechanism of ASD, where supplementation as an isolated intervention may not be sufficient. Additionally, results may vary depending on the type of probiotics used, and many studies do not specify the dose or manufacturers, which introduces variability. An important factor is gastrointestinal disorders, such as constipation and diarrhea, which can affect the absorption of probiotics, reducing their effectiveness. Although the improvement in gastrointestinal problems and the indirect impact on patient behavior are promising, the direct impact of probiotics on the core symptoms of ASD remains unproven [36,37].

## 5. Co-Occurring Feeding and Eating Disorders

The interplay of sensory sensitivity, behavioral rigidity, and gut–brain axis dysfunction predisposes individuals with ASD to a wide spectrum of specific Feeding and Eating Disorders (FEDs)—Table 2.

### 5.1. ARFID, Pica, and Rumination

Avoidant/Restrictive Food Intake Disorder (ARFID) represents the direct clinical manifestation of the sensory and behavioral rigidity. Unlike classic eating disorders driven by body image, food avoidance in ASD is predominantly motivated by sensory aversion (e.g., texture, smell) or fear of aversive consequences [38,39]. This is corroborated by recent studies indicating that the sensory and mixed subtypes of ARFID are significantly overrepresented in the ASD population [39,53]. Consequently, a “restrictive pathway” emerges where sensory hypersensitivity leads to food refusal, which is reinforced by behavioral rigidity, resulting in severe nutritional deficiencies. Additionally, behaviors such as pica and rumination are frequently observed, particularly in individuals with co-occurring intellectual disabilities, further complicating the clinical picture [40,41].

### 5.2. Anorexia Nervosa and Shared Cognitive Phenotypes

The high comorbidity between ASD and Anorexia Nervosa (AN) suggests shared neurocognitive underpinnings. The “pathogenic pathway” involves not only cognitive inflexibility and set-shifting deficits but also shared difficulties in emotion recognition and theory of mind [42,54,55,56,57]. Unlike typical AN driven primarily by body image distortion, restrictive behaviors in ASD often stem from a need for control and predictability. Crucially, the physiological state of starvation creates a self-perpetuating cycle by intensifying pre-existing autistic traits such as obsessions, rituals, and social withdrawal [58,59,60]. This presents a significant diagnostic challenge, as it remains debated in the literature whether these symptoms represent a true ASD comorbidity or secondary consequences of malnutrition that might resolve with weight restoration [61,62,63]. Consequently, this specific phenotype is associated with a poorer clinical course and resistance to standard therapeutic interventions that fail to address the underlying sensory sensitivity and cognitive rigidity [44,64,65].

### 5.3. Orthorexia Nervosa: A Manifestation of Cognitive Rigidity

Orthorexia Nervosa (ON) in individuals with ASD should be interpreted primarily through the lens of “insistence on sameness” and cognitive rigidity rather than purely as a pursuit of vitality [48]. Although clear diagnostic criteria remain debated and prevalence estimates vary widely (from 2.6% to 87.7%), the clinical overlap is significant [66,67]. Research indicates a high prevalence of autistic traits in individuals with orthorexia tendencies, suggesting shared psychopathological mechanisms [45,47]. For patients on the spectrum, rigid adherence to dietary rules (e.g., eating only “pure” foods) often serves as a maladaptive coping mechanism to impose order and predictability on their environment, differentiating it from the body-image motivations typical of the general population [7,48].

### 5.4. Binge Spectrum Disorders: The Metabolic-Emotional Pathway

While restrictive patterns are prominent, a distinct pathogenic pathway leads to overweight and obesity via Binge-Eating Disorder (BED) and, less frequently, Bulimia Nervosa [50,51]. Although bulimic behaviors (binge-purge cycles) occur in this population, they are less frequent than restrictive mechanisms and are characterized by significantly lower preoccupation with food and body shape compared to neurotypical eating disorders [51,52]. In this trajectory, the gut dysbiosis may play a causal role by disrupting the regulation of neurotransmitters involved in mood and appetite control. This biological dysregulation, combined with alexithymia and difficulties in emotional processing, predisposes individuals to “emotional eating”. As hypothesized by Wallace et al., binge-eating episodes in ASD often function as a self-stimulation strategy—a repetitive behavior aimed at providing sensory stimuli to regulate negative emotional or cognitive states [68]. Consequently, in the absence of compensatory behaviors (which are less common in ASD than in neurotypical bulimia), this “emotional eating” pathway facilitates the development of BED and contributes to the high rates of obesity observed in this population [51,69,70].

## 6. Impact on Body Weight and Overall Health

**Figure 2:**
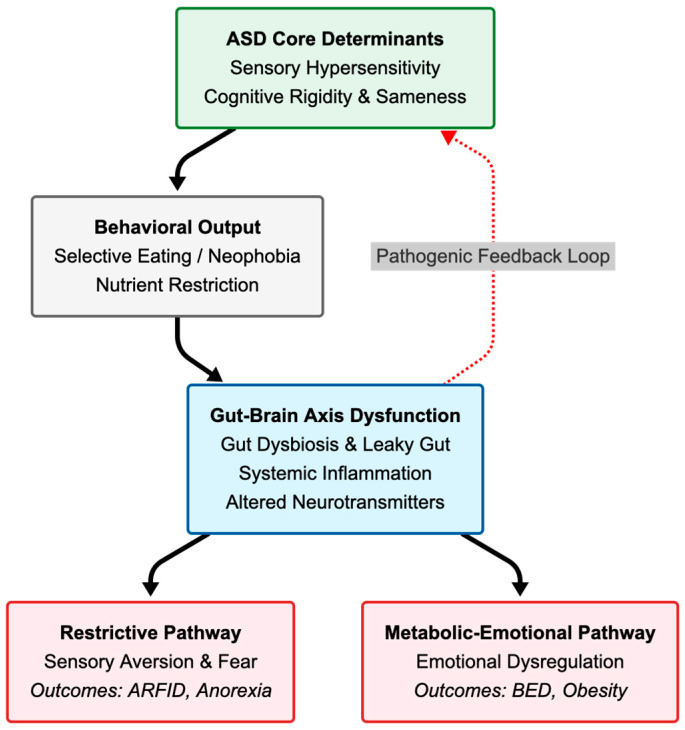
Integrated pathogenic model of co-occurring eating disorders (ED) in Autism Spectrum Disorder (ASD). Figure created by the authors.

The bimodal distribution of eating problems in ASD leads to distinct somatic risks (Figure 2). Restrictive behaviors often result in a monotonous diet, which has been observed from infancy [24,71,72]. The long-term consequences include severe micronutrient deficiencies, particularly of vitamins A, D, C, E, and the B-complex (B6, B12, folic acid), as well as essential minerals like iron, zinc, calcium, and magnesium [73,74,75]. These deficits have systemic implications: insufficient vitamin D and calcium compromise bone density, increasing fracture risk, while deficiencies in immune-modulating nutrients (zinc, vitamins A/C) heighten susceptibility to infections [73]. In extreme cases, critical conditions such as scurvy or vitamin A deficiency-related visual impairments have been reported due to severe food selectivity [74,76]. Furthermore, malnutrition can lead to oral health issues, including gingivitis and delayed wound healing [30,77,78].

Conversely, the preference for energy-dense, low-fiber foods, combined with sedentary habits and pharmacotherapy, contributes to a high prevalence of overweight and obesity, estimated at over 30% in some ASD cohorts [71,79,80]. This metabolic phenotype increases the long-term risk of cardiovascular disease and diabetes [81,82]. Additionally, chronic gastrointestinal dysfunction, manifesting as constipation or diarrhea, affects up to 70% of patients. While often linked to diet and dysbiosis, these GI symptoms can exacerbate behavioral irritability and sleep disturbances, creating a vicious cycle of somatic discomfort and behavioral dysregulation [73,75,83]. Importantly, deficiencies in omega-3 fatty acids and iron may further impair cognitive functions, such as attention and memory—domains that are fundamentally disrupted in neurodevelopmental disorders—directly impacting the child’s developmental trajectory [73,84].

## 7. Conclusions and Clinical Implications

The complex co-occurrence of Eating Disorders and Autism Spectrum Disorder is driven by a pathogenic feedback loop involving sensory hypersensitivity, behavioral rigidity, and dysfunction of the gut–brain axis.

This review demonstrates that feeding difficulties in ASD should be understood not merely as behavioral comorbidities but as the result of intricate neurobiological interactions. From a clinical perspective, this necessitates a shift in diagnostic protocols to carefully differentiate between classic eating disorders driven by body image distortion and phenotypes specific to ASD. In cases of severe restriction, such as ARFID or Anorexia Nervosa, routine screening for sensory processing disorders and gastrointestinal dysfunction is imperative, as these are often the primary drivers of food refusal [22,23,75]. Moreover, standard therapeutic interventions, such as rapid refeeding, may be ineffective or traumatic for patients with sensory defensiveness. Therefore, effective management requires a multidisciplinary strategy integrating sensory integration therapy, nutritional rehabilitation respectful of “safe foods”, and behavioral interventions targeting cognitive rigidity [73,78].

The recognition of gut dysbiosis as a mediator of behavioral symptoms also highlights new therapeutic targets. While microbiological interventions like probiotics or fecal microbiota transplantation offer promise, their routine clinical application awaits validation through longitudinal studies, such as the GEMMA project [18,28]. Ultimately, moving beyond purely behavioral management toward a holistic biopsychosocial model is essential to improve long-term health outcomes and quality of life for this population.

Finally, given the complex neurobiological underpinnings of these disorders, future clinical protocols might benefit from integrating nutritional rehabilitation with neurophysiological monitoring [5]. Tools such as quantitative electroencephalography (QEEG) and event-related potentials (ERPs) could serve as objective biomarkers to assess functional brain changes resulting from dietary interventions, while neurotherapeutic modalities like Neurofeedback may support the emotional regulation necessary to break the cycle of binge eating [85,86,87]. However, further research is needed to validate these integrative approaches in the ASD population.

## Figures and Tables

**Table 1:** Factors Influencing Eating Patterns in Individuals with ASD.

Factor	Impact on Eating Patterns	Sources
Sensory Processing Disorders	Hypersensitivity in the oral cavity leads to avoidance of specific textures, consistencies, and colors of food. Difficulties with oral hygiene (e.g., tooth brushing). Results in food selectivity and affects body weight.	[22,23]
Behavioral Rigidity and Need for Predictability	Repetitive behavioral patterns hinder the introduction of new foods. Parents often serve the same meals repeatedly, leading to limited dietary diversity, nutritional deficiencies, and social challenges.	[24,25]
Self-Injurious Behaviors and Oral Health	Self-harm in the head and neck area (e.g., head banging, biting, tooth pulling) causes oral pathologies: injuries, gingivitis, tooth loss, and microbiota imbalance. Atypical oral habits include bruxism, tongue thrusting, chewing non-food items, and regurgitation.	[14]

These factors are supported by research from Ayres et al., Xiang et al., Emond et al., D’Angelo et al. and others [22,23,24,25].

**Table 2:** Characteristics of Eating Disorders Co-occurring with ASD.

Eating Disorder	Key Features	Relationship with ASD	Source
ARFID (Avoidant/Restrictive Food Intake Disorder)	A significant reduction in the amount and variety of food consumed, leading to malnutrition; not associated with a distorted body image	Very frequent co-occurrence; the sensory subtype is most commonly observed in individuals with ASD. Among children with ARFID, 8.2% to 54.8% also have an ASD diagnosis	[38,39]
Pica	Persistent consumption of foods that are not suitable for eating for more than a month	It is more common in autistic people with concomitant intellectual disability	[1,40,41]
Rumination Disorder	Repeated regurgitation of undigested or partially digested food from the stomach	More common in autistic individuals with a co-occurring intellectual disability	[1,41]
Anorexia Nervosa	Deliberate restriction of food intake, an intense fear of gaining weight, and a distorted body image	A significant overrepresentation of autistic traits in individuals with anorexia. Co-occurrence is associated with a poorer clinical course	[2,10,42,43,44]
Orthorexia Nervosa	Pathological obsession with proper or “pure” nutrition, involving rigid dietary rules and psychosocial impairment, primarily driven by a desire for health rather than weight loss	An overlap in symptoms between the autism spectrum and ON has been noted, as well as an increased frequency of autistic traits in individuals with orthorexic tendencies.	[45,46,47,48,49]
Binge Spectrum Disorders (Bulimia, BED)	Episodes of binge eating, followed by compensatory behaviors (e.g., purging) in bulimia	Less common than restrictive mechanisms. Binge eating can be a strategy for coping with difficult emotions	[2,50,51,52]

## Abbreviations

The following abbreviations are used in this manuscript:

4EPS	4-ethylphenylsulfate
ADHD	Attention Deficit Hyperactivity Disorder
ADOS	Autism Diagnostic Observation Schedule
AN	Anorexia Nervosa
AQ-50	Autism-Spectrum Quotient, 50 questions
ARFID	Avoidant/Restrictive Food Intake Disorder
ASD	Autism Spectrum Disorder
BED	Binge-Eating Disorder
BMI	Body Mass Index
CDC	Centers for Disease Control and Prevention
DSM-5	Diagnostic and Statistical Manual of Mental Disorders, Fifth Edition
EAT-26	Eating Attitudes Test, 26 items
GEMMA	Genomic, Environmental, Microbiome, and Metabolomic Assessment
GI	Gastrointestinal
ICD-11	International Classification of Diseases, 11th Revision
ON	Orthorexia Nervosa
TD	Typically Developing
VAD	Vitamin A Deficiency
